# The Complete Genome Sequences of 87 Species of Hawks (Accipitriformes, Aves)

**DOI:** 10.56179/001c.67877

**Published:** 2023-01-09

**Authors:** Therese A Catanach, Stacy Pirro

**Affiliations:** 1 Department of Ornithology, Academy of Natural Sciences, Drexel University; 2 Biodiversity, Iridian Genomes

**Keywords:** genome, birds, hawks

## Abstract

We present the complete genome sequences of 87 species of hawks from 39 genera. Illumina sequencing was performed on genetic material from single individuals. The reads were assembled using a *de novo* method followed by a finishing step. The raw and assembled data are publicly available via Genbank.

## Introduction

Hawks (Accipitriformes) include 280 extant species in 74 genera (Lepage, Vaidya, and Guralnick 2014). We present the assembled genome sequences from 87 extant species of hawks. Tissue or toepad samples were obtained from vouchered museum specimens. More detailed information about the specimen used for each species can be found in the Biosample linked to each genome assembly in Genbank.

## Methods

DNA extraction was performed using the Qiagen DNAeasy genomic extraction kit using the standard protocol. A paired-end sequencing library was constructed using the Illumina TruSeq kit according to the manufacturer’s instructions. The library was sequenced on an Illumina Hi-Seq platform in paired-end, 2 × 150 bp format. The resulting fastq files were trimmed of adapter/primer sequence and low-quality regions with Trimmomatic v0.33 (Bolger, Lohse, and Usadel 2014). The trimmed sequence was assembled by SPAdes v2.5 (Bankevich et al. 2012) followed by a finishing step using Zanfona (Kieras, O’Neill, and Pirro 2021).

## Results and Data Availability

All data, including raw reads and assembled genome sequence, is available via Genbank.

**Table:** 

*Accipiter castanilius*	JANBPM000000000
*Accipiter cooperii*	JANBPI000000000
*Accipiter fasciatus*	JANBPN000000000
*Accipiter gentilis atricapillus*	JAOEIF000000000
*Accipiter hiogaster*	JANCIS000000000
*Accipiter melanochlamys*	JANBZU000000000
*Accipiter melanoleucus*	JANEXH000000000
*Accipiter nisus*	JANBZV000000000
*Accipiter novaehollandiae*	JANEXO000000000
*Accipiter rufitorques*	JANHGY000000000
*Accipiter rufiventris*	JANUFW000000000
*Accipiter soloensis*	JAOEIG000000000
*Accipiter superciliosus*	JAOBUD000000000
*Accipiter virgatus*	JAOSZC000000000
*Aegypius monachus*	JANUXQ000000000
*Aquila africana*	JANCMH000000000
*Aquila audax*	JANVCD000000000
*Aquila nipalensis*	JANCNU000000000
*Aquila spilogaster*	JANHGO000000000
*Aviceda subcristata*	JANVCT000000000
*Busarellus nigricollis*	JANVCM000000000
*Butastur indicus*	JANZMB000000000
*Butastur rufipennis*	JANVAC000000000
*Buteo albigula*	JANVCN000000000
*Buteo albonotatus*	JANVAB000000000
*Buteo buteo*	JANVCR000000000
*Buteo hemilasius*	JAOEIE000000000
*Buteo jamaicensis*	JANVCO000000000
*Buteo lagopus*	JAOEIH000000000
*Buteo lineatus*	JANVCS000000000
*Buteo nitidus*	JANVCP000000000
*Buteo oreophilus*	JANZYC000000000
*Buteo platypterus*	JAOBAO000000000
*Buteo regalis*	JANXJG000000000
*Buteo rufinus*	JAOYNR000000000
*Buteo swainsoni*	JANXJI000000000
*Buteo ventralis*	JANUXP000000000
*Buteogallus aequinoctialis*	JANZYB000000000
*Buteogallus coronatus*	JANZLI000000000
*Buteogallus gundlachii*	JAOEJP000000000
*Circaetus cinerascens*	JANZLZ000000000
*Circaetus gallicus*	JANZMD000000000
*Circus approximans*	JANZMF000000000
*Circus cinereus*	JANZLN000000000
*Circus hudsonius*	JANZYE000000000
*Circus macrourus*	JANZLW000000000
*Circus maillardi*	JAOEIJ000000000
*Cryptoleucopteryx plumbea*	JANZME000000000
*Elanoides forficatus*	JAOBAS000000000
*Elanus axillaris*	JANZLQ000000000
*Geranoaetus albicaudatus*	JANZLS000000000
*Geranoaetus polyosoma*	JANZYD000000000
*Geranospiza caerulescens*	JANZXY000000000
*Gyps fulvus fulvus*	JAOEID000000000
*Gyps rueppelli*	JANZYA000000000
*Haliaeetus ichthyaetus*	JAOEHU000000000
*Haliaeetus leucogaster*	JAOEIM000000000
*Haliastur indus*	JANZXX000000000
*Haliastur sphenurus*	JANZXZ000000000
*Harpagus bidentatus*	JAOBAP000000000
*Harpyhaliaetus solitarius*	JANXJE000000000
*Harpyopsis novaeguineae*	JANZLL000000000
*Henicopernis longicauda*	JAOBAQ000000000
*Hieraaetus ayresii*	JAOYMW000000000
*Hieraaetus morphnoides*	JAOXJU000000000
*Hieraaetus pennatus*	JAOEIO000000000
*Ictinia mississippiensis*	JANXJD000000000
*Leptodon cayanensis*	JANXJC000000000
*Leucopternis kuhli*	JANZLT000000000
*Leucopternis melanops*	JANZMA000000000
*Macheiramphus alcinus*	JAOEIN000000000
*Megatriorchis doriae*	JAOEIL000000000
*Melierax canorus*	JAOBAN000000000
*Melierax metabates*	JAOEIK000000000
*Melierax poliopterus*	JANZMC000000000
*Micronisus gabar*	JAOEHL000000000
*Milvus milvus*	JANZLG000000000
*Nisaetus cirrhatus*	JANXJJ000000000
*Nisaetus nipalensis*	JANZLV000000000
*Pandion haliaetus*	JANZLM000000000
*Parabuteo unicinctus*	JAOBAR000000000
*Pernis celebensis*	JAOYMY000000000
*Polyboroides typus*	JANZLO000000000
*Rostrhamus sociabilis*	JANXJF000000000
*Spilornis abbotti*	JANXJH000000000
*Spizaetus melanoleucus*	JANZXW000000000
*Spizaetus ornatus*	JANZLU000000000
